# Spotlight on HIV-1 Nef: SERINC3 and SERINC5 Identified as Restriction Factors Antagonized by the Pathogenesis Factor

**DOI:** 10.3390/v7122970

**Published:** 2015-12-19

**Authors:** Oliver T. Fackler

**Affiliations:** 1 Department of Infectious Diseases, Integrative Virology, University Hospital Heidelberg, Im Neuenheimer Feld 324, 69120 Heidelberg, Germany; oliver.fackler@med.uni-heidelberg.de; Tel.: +49-6221-561322; Fax: +49-6221-565003; 2 German Center for Infection Research, Heidelberg University, 69120 Heidelberg, Germany

**Keywords:** HIV-1, restriction factors, Nef protein, virion infectivity, SERINC3, SERINC5

## Abstract

The Nef protein is an accessory gene product encoded by human immunodeficiency virus types 1 and 2 (HIV-1/-2) and simian immunodeficiency virus (SIV) that boosts virus replication in the infected host and accelerates disease progression. Unlike the HIV-1 accessory proteins Vif, Vpr and Vpu, Nef was, until recently, not known to antagonize the antiviral activity of a host cell restriction factor. Two recent reports now describe the host cell proteins serine incorporator 3 and 5 (SERINC3 and SERINC5) as potent inhibitors of HIV-1 particle infectivity and demonstrate that Nef counteracts these effects. These findings establish SERINC3/5 as restrictions to HIV replication in human cells and define a novel activity for the HIV pathogenesis factor Nef.

## 1. Introduction

**Figure 1:**
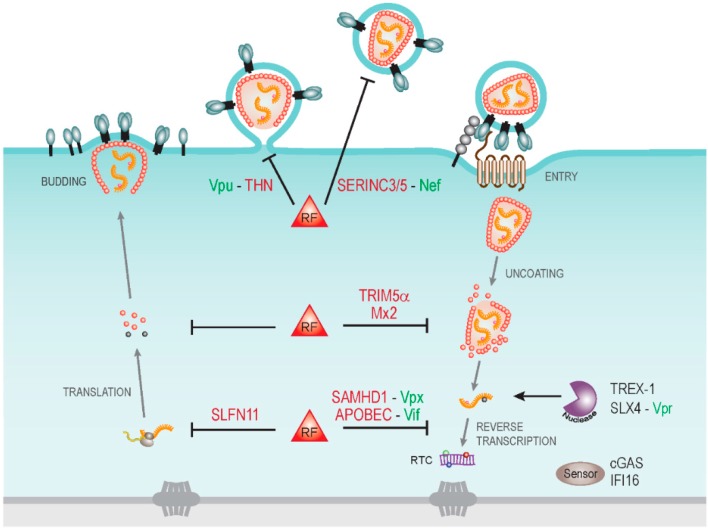
Cytoplasmic host cell restrictions to human immunodeficiency virus types 1 (HIV-1) infection and virally encoded antagonists. Schematic depiction of the HIV-1 life cycle in the cytoplasm of a target cell with some restriction factors (RF) and their viral antagonists indicated. Early post entry steps of HIV-1 replication are particularly targeted by host cell restriction factors including TRIM5α and Mx2 that recognize viral cores and may affect their stability. Uncoating of viral capsids renders viral RNA genomes accessible to host cell nucleases such as TREX1 that reduce innate immune recognition by the host cell and thus benefit HIV replication. Such a strategy may be exploited by the HIV-1 Vpr protein that activates the SLX4 endonuclease complex. Reverse transcription of viral RNA genomes into DNA is targeted by the cytidine deaminase activity of the apolipoprotein B mRNA editing enzyme, catalytic polypeptide-like (APOBEC) proteins and the triphosphohydrolase SAMHD1, which are antagonized by the viral proteins Vif and Vpx (Vpx is only encoded bythe human immunodeficiency virus type 2 (HIV-2) and the simian immunodeficiency virus (SIV) and is lacking in HIV-1). Reverse transcription products are recognized by cytoplasmic DNA sensors such as cGAS and Ifi-16 to trigger innate immune responses. During virus production, translation of viral mRNA can be restricted by Schlafen11 (SLFN11). At the late stages of particle production, viral progeny is trapped at the cell surface by tetherin (THN), a restriction antagonized by Vpu. The infectivity of released particles can be significantly compromised by the newly described restriction factors serine incorporator 3 and 5 (SERINC3/5) and their antiviral activity is antagonized by Nef.

As a complex retrovirus, human immunodeficiency virus type 1 (HIV-1) encodes for the classical structural retroviral proteins Gag, Pol and Env as well as for the regulatory proteins Tat and Rev, but also for so called accessory proteins (Vif, Vpr, Vpu and Nef in the case of HIV-1). While structural and regulatory proteins are essential for HIV replication irrespective of the cellular context, accessory genes encode for proteins that can be dispensable for HIV-1 spread in *ex vivo* cell line cultures as they, among other functions, mediate the interaction of infected cells with the host immune system. As an example for such activity, Nef and Vpu facilitate evasion of HIV-1 infected cells from recognition and thus lysis by cytotoxic T cells and natural killer cells [1,2,3,4]. Over the past decade it emerged that a general theme of HIV accessory protein function is the counteraction of host cell barriers against retroviral replication that are referred to as restriction factors and represent an important arm of the cell-autonomous host defense system [5]. With even more restriction factors likely to be discovered, currently known host cell restrictions are already placed all along the HIV-1 life cycle (Figure 1). Considering the antiviral potency of some restriction factors, HIV-1 depends on active principles to overcome these barriers for efficient replication in target cells with robust restriction factor expression and many of these mechanisms depend on accessory protein function. In HIV-1, this paradigm was first established for the apolipoprotein B mRNA editing enzyme, catalytic polypeptide-like 3G (APOBEC3G), a cytidine deaminase that limits HIV replication by elevating the mutation rate during reverse transcription of incoming RNA genomes into DNA [6]. APOBEC3G is efficiently antagonized by HIV-1 Vif by targeting it for degradation and thereby preventing its incorporation into virus particles [5]. Vpu in turn counteracts the restriction to HIV particle release imposed by the restriction factor tetherin (also referred to as BST-2 or CD317) [7,8], presumably by lateral displacement away from virus budding sites [9]. Vpr antagonizes a macrophage-specific restriction to limit expression of Env and production of infectious progeny that awaits identification of the molecules involved [10,11]. Vpr also reduces production of antiviral cytokines by innate immune sensing through the premature activation of the SLX4 endonuclease complex [12]. Among HIV-1 accessory proteins, Nef remained the only member for which antagonism of a host cell restriction factor had not been identified and Nef was hence considered an orphan restriction factor antagonist.

## 2. HIV-1 Nef: A Multifunctional Adaptor Protein

Interest in the molecular mechanisms of Nef function was initially triggered by the observation that simian immunodeficiency virus (SIV) or HIV variants that lack expression of functional Nef proteins replicate with reduced efficiency in the infected host and lead to significantly delayed clinical progression [13,14,15]. While these studies clearly defined Nef as an important parameter for lentiviral pathogenesis, unraveling the relevant molecular mechanisms was hampered by the multitude of effects that can be observed as consequence of Nef expression in HIV target cells as well as the plethora of low affinity interactions in which the viral protein engages with host cell proteins [16,17,18]. Taken together these studies suggest that Nef acts as protein adaptor without enzymatic activity to hijack central host cell transport and signal transduction pathways and to optimize virus spread in the infected host. An important aspect of these activities is the re-routing of transmembrane receptors (such as the HIV entry receptor CD4 or major histocompatibility complex (MHC)-I molecules) as well as peripheral membrane proteins (such as Src family kinases) from the plasma membrane to intracellular membrane compartments. In addition, Nef also impairs host cell actin dynamics and motility, drives the release of extracellular vesicles, and has been implicated in the regulation of cell survival [2,19,20,21,22,23,24,25,26,27]. While some of these effects have been characterized in great detail at the cellular and molecular level, their relevance and relative contribution to HIV pathogenesis largely remain to be defined.

## 3. Enhancement of Virion Infectivity by Nef

Early characterization of *nef*-deficient HIV-1 variants with wild-type HIV-1 revealed that Nef elevates the infectivity of virus particles [28], an activity that provided an intuitive link to the observation that the viral protein elevates viral titers in infected host by several orders of magnitude. The effect of Nef on virion infectivity was detected in single round infection assays in which wild-type HIV-1 was two-to-tenfold more infectious than the HIV-1∆*nef* counterpart (reviewed in [29]). This effect required the presence of Nef in the producer but not the target cell or the virion itself, and was due to an effect on the infectivity of HIV-1 particles and not the amount of particles released from the producer cells [30,31,32,33]. Which step in the viral life cycle is facilitated by Nef remained somewhat controversial: most studies detected a positive effect of Nef on early post entry events without enhancement of fusion, other studies reported a mild effect of Nef on the fusion of HIV-1 particles with target cells (reviewed in [29]).

A caveat for the relevance of the Nef effect on infectivity was that it was relatively mild (two-to-tenfold fold depending on the producer cell used). This changed when Pizzato and Göttlinger described a clone of Jurkat E6.1 cells that, when used for virus production, revealed an up to 100 fold enhancement of virion infectivity by Nef [34,35]. In this system, the effect of Nef on virion infectivity depended on the GTPase dynamin 2, clathrin and the AP-2 adaptor complex, indicating that Nef may affect virion infectivity by modulating host cell endocytic trafficking [34,36].

## 4. Identification of serine incorporator 3 and 5 (SERINC 3/5) as Nef-Sensitive Restriction Factors

Following the definition of the potent Nef-mediated infectivity enhancement of virions produced from this Jurkat E6.1 clone, Göttlinger and Pizzato independently pursued the search for the underlying molecular mechanisms. A first interesting clue came from a study that identified the Moloney murine leukemia virus (MLV) protein glycoGag as retroviral protein that is unrelated to Nef but exhibits similar infectivity enhancement [37]. GlycoGag could substitute for Nef and contamination with MLV, and thus expression of glycoGag, accounted for the low magnitude of the Nef effect on HIV-1 infectivity in some of the cell lines used to study this phenomenon. Nevertheless, endogenous expression of glycoGag could not explain all producer-cell dependent differences in the effect of Nef on virion infectivity and the Pizzato and Göttlinger laboratories started to search for host cell factors that determine the impact of Nef. In a remarkable coincidence, distinct experimental strategies (proteomics of HIV particles produced in absence or presence of Nef *vs.* expression profiling of Nef-sensitive and Nef-insensitive producer cells) resulted in the simultaneous identification of the proteins SERINC3 and SERINC5 as potent inhibitor of HIV particle infectivity that are counteracted by Nef [38,39].

SERINC3 and SERINC5 are members of the serine incorporator (SERINC) protein family that is conserved from yeast to mammals and contains five members that are predicted to contain 10–12 transmembrane domains [40]. SERINC protein function has not been studied in great detail and essentially relies on a single report by Inuzuka and colleagues [39]. In this study, several SERINC proteins were demonstrated to facilitate the incorporation of serine in the biosynthesis of sphingolipids and phosphatidylserine when ectopically expressed in *E. coli*, yeast and COS-7 cells. SERINC family members share over 30% of amino acid identity, but only SERINC3 and SERINC5 displayed antiviral activity and primary Nef alleles displayed differential efficacy in the antagonism of SERINC3 or SERINC5. This selectivity among SERINC family members, but also among viral antagonists, will certainly be helpful in identifying the molecular determinants that mediate restriction and antagonism.

In addition to identifying SERINC3/5 as novel anti-HIV restriction factors that are antagonized by Nef, the studies by Rosa *et al.* [38] and Usami *et al.* [39] provide first important insights into the mechanisms of restriction and antagonism. By knock-down/knock-out and overexpression approaches, they clearly establish that SERINC3/5 are necessary but also sufficient for the HIV particle infectivity restriction. The observation that expression of SERINC3/5 implements the infectivity restriction in 293T cells that otherwise do not limit particle infectivity implies that SERINC3/5 either act largely independently of the cellular context or rely on highly conserved cellular pathways. How then does SERINC3/5 impair the infectivity of HIV-1 particles? Both studies observed that when produced in the absence of Nef, HIV-1 particles incorporate significant amounts of the restriction factor. Using delivery of a virion-incorporated reporter molecule (Vpr-Blam) to the cytoplasm of target cells as a measure of virion-target cell fusion, SERINC5 appears to significantly impair the fusogenicity of HIV-1 particles. However, Rosa *et al.* describe that this effect on fusion may not explain all effects of SERINC3/5 on virion infectivity, suggesting that also early post entry steps may be affected. The papers by Rosa *et al.* [38] and Usami *et al.* [39] also observe that the presence of Nef in the producer cell markedly reduced virion-incorporation of SERINC3/5. As judged by steady-state cell surface levels and subcellular distribution, this effect of Nef is paralleled by a redistribution of the restriction factor from the plasma membrane into an intracellular Rab7-postive membrane compartment. These effects of Nef on the subcellular localization of SERINC3/5 were confirmed by a recent study that determined the impact of Nef on the cell surface proteome of HIV-1 infected T lymphocytes [41] and identified SERINC 3/5 as targets whose cell surface exposure is reduced in the presence of Nef. Since AP-2 expression was found to be required for Nef-mediated antagonism of SERINC5 [38], these findings result in a model in which the presence of SERINC3/5 in HIV particles limits their ability to fuse with target cells. In this scenario, Nef antagonizes this antiviral activity by triggering a redistribution of the restriction factor to an endocytic compartment, thereby excluding its incorporation into virus particles.

## 5. Open Questions and Future Directions

The milestone papers by Rosa *et al.* [38] and Usami *et al.* [39] define SERINC3 and SERINC5 as potent antiviral host cell factors that are antagonized by HIV-1 Nef. This important discovery raises a number of fascinating new questions that will undoubtedly re-intensify efforts to unveil the mechanisms by which Nef drives lentiviral pathogenesis. A key aspect of these studies will be on the cellular context in which SERINC3/5 restriction and antagonism by Nef is physiologically relevant. The paper by Usami and colleagues [39] provided evidence that monocyte-derived macrophages (MDMs) express elevated levels of SERINC3 and SERINC5 mRNA and that silencing of these factors enhances the infectivity of virions produced from these cells. Similarly, knock-out of SERINC5 in peripheral blood mononuclear cells (PBMC) significantly enhanced the infectivity of *nef*-negative HIV-1 in the study by Rosa *et al.* [38]. These results demonstrate that the SERINC3/5 restriction occurs in relevant primary HIV target cells *ex vivo*. It will now be important to assess under which physiological conditions SERINC3/5 expression and/or activity reach the levels required for restriction of HIV replication. Based on first evidence provided in these two papers, SERINC3/5 mRNA expression is not induced by interferon α, interferon ß, lipopolysaccharide or phytohemagglutinin and it remains thus unclear how SERINC3/5 is regulated. Future analyses will have to assess SERINC3/5 expression on the protein level to define the per cell concentrations of these restriction factors required for interference with virion infectivity. Since detection of endogenous SERINC3/5 proteins is technically challenging, the development of robust and reliable tools for endogenous protein detection will be a prerequisite to address these important issues.

Another important aspect of the study by Usami and colleagues [39] was to test whether in addition to the effects on virion infectivity in a single round of infection, counteraction of SERINC 3/5 by Nef also impacts HIV spread over multiple replication rounds. Using Jurkat E6.1 cells, they demonstrated that Nef provides HIV with a significant replication advantage in the presence of the restriction factors while *nef*-negative HIV replicates as efficiently as wild-type HIV-1 when SERINC3/5 expression is ablated. However, in the presence of the restriction factors, replication of HIV-1∆*nef* was delayed but not suppressed as might have been suggested by the very potent reduction to single rounds of HIV-1 infection. These replication kinetics resembles those observed for HIV-1 wild-type and ∆*nef* in primary T lymphocyte, *ex vivo* tonsil histoculture, or MDM cultures [42,43,44,45,46] and would be consistent with a potent block to initial rounds of replication that is less severe in subsequent replication rounds. In T lymphocyte cultures, cell-associated modes of virus transmission significantly contribute to HIV spread [47]. While Nef does not affect frequency and overall organization of the cell-cell contacts required for virus transmission [48], Nef facilitates recruitment of HIV-1 Gag to the plasma membrane to enhance productive cell-associated HIV-1 transmission [49]. The effect of Nef on a single round of cell-associated infection (2–3 fold) was much less pronounced than that on cell-free infection with SERINC3/5-positive HIV-1 (approx. 100 fold). However, these cell-to-cell transmission assays were not conducted in the presence of high levels of SERINC3/5 and it will thus be interesting to address the role of SERINC3/5 restriction and Nef antagonism in the context of cell-associated modes of HIV transmission.

As discussed above, the data presented by Rosa *et al* [38] and Usami *et al* [39] are consistent with the proposed hypothesis that the antiviral activity of SERINC3/5 is exerted within the virion and that re-routing of the restriction factors by Nef in the producer cell leads to exclusion of SERINC 3/5 from HIV-1 particles, thereby alleviating their antiviral effect. Experiments designed to directly challenge whether the observed correlation between SERINC3/5 localization and antiviral activity provides an essential mechanistic link will be required and may lead to additional, not mutually exclusive hypotheses. The key challenge will be to unravel the principle by which SERINC3/5 inhibit virion infectivity. Based on the very limited knowledge about SERINC protein function, it is tempting to speculate that their antiviral activity is linked to the ability to facilitate the incorporation of serine into biosynthetic pathways for phosphatidylserine and sphingolipids [40]. In line with this hypothesis, HIV particles are enriched in both these lipid classes, their specialized lipid composition is an essential parameter for infectivity, and Nef moderately affects the lipid composition of HIV-1 particles when produced from MT-4 cells that lack functional expression of SERINC3/5 [50,51]. However, whether and how endogenous SERINC proteins affect lipid biosynthesis or transport in mammalian cells and thus possibly HIV-1 particle composition produced from them remains to be investigated. Alternatively, SERINC3/5 protein may directly interfere with HIV infectivity, e.g., by steric hindrance of the fusion machinery or by disruption of particle architecture. In this regard it is noteworthy that the effect of Nef on HIV-1 particle infectivity is linked to the HIV Env variant used and that determinants in the V1 and V2 region of Env define whether Env proteins are sensitive to infectivity enhancement by Nef [52]. This selectivity of glycoproteins in their responsiveness to Nef is reminiscent of the long-standing observation that pseudotyping of HIV-1 particles with vesicular stomatitis virus glycoprotein but not MLV Env renders HIV-1 particles insensitive to infectivity enhancement by Nef [53,54]. The molecular principles underlying this functional interplay between Nef and Env likely hold important clues for our understanding of the SERINC3/5 particle infectivity restriction, but also for the mechanisms by which Nef antagonizes it.

Host cell restriction to retroviruses often operate in a highly species specific manner and while antiviral activity of restriction factors tends to be conserved in evolution, viral antagonists are often species-adapted [55]. With Nef from HIV-1 but also SIVmac239 as well as MLV glycoGag, several entirely independent mechanisms for antagonism of human SERINC3/5 emerged in retroviral evolution and more likely remain to be identified. Of note, the discovery of SERINC3/5 adds, in addition to tetherin, a second important restriction that acts at the late stages of the viral life cycle. Nef and Vpu, the respective HIV-1 antagonists of SERINC3/5 and tetherin, diversified from SIV lineages that do not encode for a Vpu protein and integrate both antagonistic activities in the respective Nef protein [55]. It will be interesting to study whether the need for counteraction of two restriction factors during the final steps of virion biosynthesis drove the evolution into separate *nef* and *vpu* genes. Finally, future studies will reveal whether the activity of SERINC3/5 is limited to retroviruses or if other virus families or pathogen classes are also affected by this host barrier.

## 6. Conclusions

The discovery of SERINC3/5 as inhibitors of HIV-1 particle infectivity identifies a novel cell-intrinsic line of defense against retroviruses and unveils the long-sought host cell factors antagonized by HIV-1 Nef. These findings will spur intense efforts targeted at addressing the precise molecular mechanisms of restriction and antagonism, the patho-physiological implications of these new virus-host interactions, and their evolution.
